# Prevalence of Sexual Dysfunction with Chronic Prostatitis/Chronic Pelvic Pain Syndrome (CP/CPPS): An Updated Systematic Review and Meta-Analysis

**DOI:** 10.3390/medicina61061110

**Published:** 2025-06-19

**Authors:** Saad Alshahrani, Basem A. Fathi, Tamer A. Abouelgreed, Ashraf El-Metwally

**Affiliations:** 1 Department of Surgery, Division of Urology, College of Medicine, Prince Sattam bin Abdulaziz University, Al-Kharj 11942, Saudi Arabia; 2 Department of Urology, Faculty of Medicine, Al-Azhar University, Cairo 11884, Egypt; basemabdalla.8@azhar.edu.eg (B.A.F.); tamerali.8@azhar.edu.eg (T.A.A.); 3 College of Public Health and Health Informatics, King Saud bin Abdulaziz University for Health Sciences, Riyadh 11426, Saudi Arabia; elmetwally.ashraf@outlook.com; 4 King Abdullah International Medical Research Center, Riyadh 11481, Saudi Arabia

**Keywords:** urogenital, prostatitis, pelvic pain, erectile dysfunction, urological epidemiology, burden

## Abstract

*Background and Objectives*: Chronic prostatitis/chronic pelvic pain syndrome (CP/CPPS) is a common condition linked to substantial urogenital symptoms, notably sexual dysfunction. This meta-analysis sought to determine the overall prevalence of sexual dysfunction in men with CP/CPPS, considering the four primary categories: desire, arousal, orgasm, and pain disorders. *Materials and Methods*: A systematic literature review, following MOOSE guidelines, was performed across four electronic databases (PubMed, Embase, Web of Science, and Google Scholar) for the period from January 2000 to 2025. The review included observational studies reporting the prevalence of sexual dysfunction in men with CP/CPPS. Data extraction and quality assessment were conducted independently by two reviewers. A random-effects model was used to calculate the pooled prevalence estimates and 95% confidence intervals. Heterogeneity was evaluated using I^2^, τ^2^, and Chi-squared tests, while publication bias was assessed via funnel plot asymmetry and Egger’s test. *Results*: The meta-analysis incorporated data from 26 studies, representing a total of 20,127 participants. The pooled prevalence of overall sexual dysfunction was 59% (95% CI: 34–81%; I^2^ = 98%) across six studies and 5333 participants. Pooled erectile dysfunction (ED) prevalence was 34% (95% CI: 26–42%; I^2^ = 99%) across 24 studies with 20,127 participants, whereas pooled prevalence for premature ejaculation (PE) was 35% (95% CI: 22–49%; I^2^ = 98%) across 10 studies with 13,686 participants. Significant heterogeneity was observed across all analyses (I^2^ > 98%). Funnel plot analysis suggested potential asymmetry, but Egger’s test was non-significant (*p* = 0.7034). *Conclusions*: This meta-analysis confirms the high prevalence of sexual dysfunction, including ED and PE, in men with CP/CPPS, providing a comprehensive estimate of its burden. The substantial heterogeneity observed underscores the need for further research to identify contributing factors and develop targeted interventions.

## 1. Introduction

Chronic prostatitis/chronic pelvic pain syndrome (CP/CPPS), as defined by the European Association of Urology (EAU) guidelines, is a prevalent urological condition characterized by pelvic pain, urinary symptoms, and/or sexual dysfunction, significantly impacting men’s quality of life [1,2]. CP/CPPS represents a spectrum of disorders, encompassing various subtypes with distinct clinical presentations and underlying pathophysiological mechanisms. While the exact etiology remains incompletely understood, contributing factors may include age, smoking status, holding urine, infection, inflammation, neuromuscular dysfunction, and psychological factors [3,4,5]. This complexity in its origin and presentation poses significant challenges in diagnosis and management [6]. Among the diverse symptoms associated with CP/CPPS, sexual dysfunction is a particularly distressing manifestation, affecting a substantial proportion of affected men [7,8].

Sexual dysfunction encompasses four main domains: desire, arousal, orgasm, and pain disorders [9,10]. Among the clinical manifestations associated with CP/CPPS, erectile dysfunction (ED) and premature ejaculation (PE) are two frequently reported sexual symptoms, each representing distinct clinical entities with specific diagnostic criteria [9,11]. These sexual difficulties can have profound psychological and social consequences, contributing to anxiety, depression, relationship problems, and diminished self-esteem, with a severe impact on quality of life and economic burden [12,13]. The impact of CP/CPPS, regardless of etiology, extends beyond individual well-being, often affecting partners and overall relationship satisfaction [14]. A thorough understanding of the prevalence and two frequently reported symptoms of sexual dysfunction affecting men with CP/CPPS is critical for the development of effective interventions and the improvement of their overall health and quality of life.

Previous reviews have investigated the effects of CP/CPPS on sexual dysfunction, reporting a wide range of prevalence rates, but these reviews have either included a limited number of studies [15] or the reviews are outdated [16] and need to be updated with the current and more recent burden of CP/CPPS-related sexual dysfunction. Moreover, the variability in reported prevalence from prior reviews can be attributed to several factors, including differences in the study populations (e.g., age, CP/CPPS subtype, comorbidities), the tools used to assess sexual dysfunction, and variations in the study design [15,16]. These inconsistencies make it challenging to draw definitive conclusions about the true burden of sexual dysfunction in men with CP/CPPS. Furthermore, individual studies may lack the statistical power to detect subtle but clinically meaningful associations.

CP/CPPS is a prevalent and complex urological condition that significantly impacts quality of life, particularly due to its strong association with sexual dysfunction. Previous studies and reviews have demonstrated inconsistent estimates of the prevalence of sexual dysfunction among men with CP/CPPS, likely due to variations in diagnostic criteria, study populations, and assessment tools [15,16]. Moreover, earlier reviews may not have comprehensively included newer studies that have emerged in recent years [15,16]. Given the growing recognition of sexual dysfunction as a central concern in CP/CPPS management, an updated systematic review and meta-analysis are warranted to provide more accurate, evidence-based prevalence estimates. A meta-analysis, combining data from multiple studies, offers a powerful approach to address these limitations. By pooling data from various studies, a meta-analysis can increase statistical power to more accurately estimate the prevalence of sexual dysfunction in men with CP/CPPS. It can also help to explore the potential sources of heterogeneity and identify factors that may influence the observed variability in prevalence rates. Furthermore, a meta-analysis can highlight gaps in the existing literature and inform future research directions.

This meta-analysis systematically assessed the prevalence of overall sexual dysfunction (desire, arousal, orgasm, and pain disorders), as well as the prevalence of ED and PE as individual conditions in men with CP/CPPS. The objectives were to estimate the pooled prevalence of each of these dysfunctions. This quantitative synthesis aims to improve our understanding of the burden of sexual dysfunction in CP/CPPS and inform clinical practice and future research.

## 2. Materials and Methods

### 2.1. Data Sources and Search Strategy

This systematic review and meta-analysis were conducted in accordance with the Meta-analysis of Observational Studies in Epidemiology (MOOSE) guidelines [17]. A comprehensive and systematic search was performed to identify all the relevant studies reporting on the prevalence of sexual dysfunction in men diagnosed with chronic prostatitis/chronic pelvic pain syndrome (CP/CPPS). We searched four electronic databases, PubMed, Embase, Web of Science, and Google Scholar, covering the period from January 2000 to January 2025. The starting point was chosen to capture the literature published after the introduction of the NIH classification of prostatitis in 1999, which provided a standardized framework for CP/CPPS definitions and subtypes. The search process was iterative and conducted in stages to ensure the inclusion of the most recent and relevant evidence. Initial searches began in mid-2024 and were updated through January 2025. The final search dates were 15 January 2025 for PubMed, Embase, and Web of Science and 16 January 2025 for Google Scholar.

The search strategy was systematically developed using the PICO (Population, Intervention, Comparison, and Outcome) framework, a widely accepted structure for formulating research questions and designing robust search queries for systematic reviews [18]. While the “Intervention/Exposure” and “Comparison” components are not directly applicable in prevalence studies—where the primary aim is to assess the occurrence of a condition—the PICO model remains a valuable tool for clarifying the core concepts of the research question. In this review, the Population was defined as men diagnosed with chronic prostatitis/chronic pelvic pain syndrome (CP/CPPS). The primary Outcome was sexual dysfunction, encompassing desire disorders, arousal issues, orgasmic problems, and pain-related sexual disorders. Additionally, two common symptom-specific outcomes—erectile dysfunction (ED) and premature ejaculation (PE)—were analyzed as key domains of sexual dysfunction. Search terms were constructed by combining Medical Subject Headings (MeSH) and free-text keywords representing these core domains. For example, a typical PubMed search string included the following: (“prostatitis” [MeSH Terms] OR “CPPS” [Title/Abstract]) AND (“sexual dysfunction” [MeSH Terms] OR “erectile dysfunction” [MeSH Terms] OR “premature ejaculation” [MeSH Terms]) AND (“prevalence” [MeSH Terms] OR “epidemiology” [MeSH Terms]); as shown in Table 1. To ensure completeness, we also performed manual screening of the reference lists of all eligible full-text articles and the relevant narrative or systematic reviews. No language restrictions were applied during the search, and non-English studies were included if sufficient data could be extracted from the English abstract or translation. A full list of database-specific search strategies is provided in Supplemental Table S1.

### 2.2. Study Selection and Screening

A rigorous, two-stage screening process was implemented to identify the studies eligible for inclusion in this meta-analysis. Initially, two independent reviewers screened the titles and abstracts retrieved from the systematic search using pre-defined inclusion and exclusion criteria. Studies were included if they employed an observational study design, such as cross-sectional, cohort, or case series methodologies; were original research articles published in English; and reported sufficient data to estimate the prevalence of sexual dysfunction in men with CP/CPPS. This could be achieved either by providing the total number of participants and the number affected or by directly reporting prevalence estimates. Eligible studies were also required to specifically report prevalence data for at least one of the following outcomes: overall sexual dysfunction, erectile dysfunction (ED), or premature ejaculation (PE). ED was defined as the inability to achieve or maintain an erection sufficient for satisfactory sexual activity, often indicated by an International Index of Erectile Function (IIEF) score of 21 or less. For this review, sexual dysfunction was considered a broad category encompassing decreased sexual desire, arousal difficulties, orgasmic disorders, and pain-related sexual dysfunction. Studies were excluded if they focused solely on a specific subtype of CP/CPPS without providing generalizable prevalence data, were editorials, commentaries, reviews, or abstracts lacking original data, or presented data from overlapping populations already included in other studies. Furthermore, studies that did not independently define and report CP/CPPS and sexual dysfunction (for example, those reporting combined categories such as “prostatic diseases and sexual dysfunction”) or lacked sufficient data to calculate prevalence were also excluded. The full texts of all the potentially eligible studies were retrieved and reviewed by the same two independent reviewers to confirm adherence to the inclusion criteria. Any disagreements were resolved through discussion, and when consensus could not be reached, a third reviewer was consulted.

### 2.3. Data Extraction

Two reviewers independently extracted data from each included study using a standardized data extraction form. This form was designed to capture key study characteristics, including author name, year of publication, study location, study design, sample size, and the age range of participants. In addition, detailed information regarding the assessment of sexual dysfunction was collected, such as the specific instruments used (e.g., validated questionnaires), the definitions applied for each outcome, and criteria for diagnosis. The prevalence data for each sexual dysfunction outcome—overall sexual dysfunction, erectile dysfunction (ED), and premature ejaculation (PE)—were recorded, including the number of affected men and the total number of participants per study. These data were used to compute prevalence proportions and their corresponding 95% confidence intervals. Discrepancies in extracted data between the two reviewers were resolved through discussion. In cases where consensus could not be reached, a third reviewer was consulted. Once finalized, all extracted data were compiled into a master database for statistical analysis in the meta-analysis.

### 2.4. Quality of Studies and Risk of Bias

The reporting quality of the included observational studies was assessed using the STROBE (Strengthening the Reporting of Observational Studies in Epidemiology) checklist, a 22-item tool designed to evaluate the completeness and clarity of reporting in observational studies [19]. The STROBE checklist (Supplemental Table S2) encompasses the key elements of a research manuscript, spanning from the title and abstract to the discussion and funding information, which allowed for an assessment of the reporting quality of the included studies [19]. Specifically, the assessment covered domains such as the clarity and balance of the title and abstract (item 1), the presence of a well-defined background and rationale (item 2), and a clearly stated objective (item 3) in the introduction. The description of the study design (item 4), setting and location (item 5), and eligibility criteria (item 6) were also evaluated. Furthermore, the assessment included the clear definition of variables, including diagnostic criteria, outcomes, exposures, and potential confounders (item 7), along with details on data sources and measurement methods (item 8). The reporting of efforts to address potential biases (item 9), the justification of study size (item 10), and the description of statistical methods, including those for controlling confounding (items 11 and 12), were also considered. The reporting of the participant numbers and characteristics (items 13 and 14), outcome events (item 15), confounder-adjusted risk estimates (item 16), subgroup analyses (item 17), and a summary of key results (item 18) were part of the evaluation. Finally, the assessment included the discussion of study limitations (item 19), a cautious interpretation of results (item 20), a discussion of generalizability (item 21), and a description of the funding sources (item 22).

### 2.5. Assessment of Certainty in the Body of Evidence

The certainty (or confidence) in the body of evidence for each outcome was assessed using the Grading of Recommendations, Assessment, Development, and Evaluation (GRADE) approach [20], which provides a transparent and structured process for rating the quality of evidence in systematic reviews and meta-analyses. The GRADE framework considers five key domains that can lead to downgrading the certainty of evidence: risk of bias, inconsistency (statistical heterogeneity), indirectness, imprecision, and publication bias. For each outcome—overall sexual dysfunction, erectile dysfunction (ED), and premature ejaculation (PE)—we systematically evaluated these domains to determine the strength and reliability of the synthesized findings.

Since all the included studies in this review were observational in nature, the initial certainty of the evidence for each outcome was rated as “low” in accordance with GRADE guidance. This starting point reflects the greater susceptibility of non-randomized studies to confounding and bias. However, the certainty rating could be downgraded further if serious or very serious concerns were identified within any of the five domains. Conversely, the certainty could also be upgraded if criteria, such as a large effect size, evidence of a dose–response relationship, or all plausible confounding reducing the demonstrated effect, were met—although these conditions are rare in prevalence studies.

The risk of bias was assessed at the study level using validated tools (as described in Section 2.4) and then summarized across studies for each outcome. If the majority of the contributing studies were deemed to have a high or unclear risk of bias, the certainty was downgraded accordingly. Inconsistency was evaluated by examining the degree of statistical heterogeneity using the I^2^ statistic. I^2^ values above 75% were considered indicative of substantial heterogeneity, which warranted downgrading unless explained by identifiable sources. Indirectness was considered by evaluating the population, setting, and outcome measures of the included studies. As all studies directly addressed the review question, there were no major concerns related to indirectness. Imprecision was judged based on the width of the 95% confidence intervals around the pooled prevalence estimates. Wide intervals indicating uncertainty about the true prevalence led to downgrading. Publication bias was assessed through the visual inspection of funnel plots and statistical tests such as Egger’s test. Evidence of asymmetry or small-study effects raised concerns and contributed to further downgrading.

Based on the cumulative assessment of these domains for each outcome, we assigned a final certainty ranging from high to very low—which is presented in the GRADE summary of findings table. This assessment helps contextualize the reliability of our pooled estimates and informs the interpretation of findings and implications for clinical practice and future research.

### 2.6. Statistical Analysis

All statistical analyses were conducted using R (version 4.2.2) with the meta package [21]. A random-effects model was chosen a priori for all meta-analyses to account for the anticipated methodological and clinical heterogeneity among the included observational studies [22]. Variations were expected due to differences in population demographics, diagnostic criteria, assessment tools for sexual dysfunction, and study settings. The random-effects model is more conservative than the fixed-effects model, as it assumes that the true effect size varies between studies rather than being fixed across all studies. For each outcome—overall sexual dysfunction, erectile dysfunction (ED), and premature ejaculation (PE)—we computed pooled prevalence estimates and 95% confidence intervals (CIs) using a generalized linear mixed model (GLMM), implemented through the metaprop function in the meta package. This approach is particularly appropriate for proportion and binary data and provides improved handling of between-study variability and heterogeneity by modeling random effects through a logistic regression framework with study-specific intercepts [23]. The logit transformation was applied to stabilize the variance across studies prior to pooling, and the back-transformed proportions were reported for interpretability. Statistical heterogeneity was assessed using the I^2^ statistic, which quantifies the proportion of variation in prevalence estimates that is due to heterogeneity rather than the sampling error [24]. We also calculated tau-squared (τ^2^), a measure of between-study variance, and conducted the Cochran’s Q (Chi-squared) test, with a *p*-value < 0.05 indicating statistically significant heterogeneity [25]. The visual inspection of funnel plots was used to evaluate potential publication bias [26]. In addition, Egger’s regression test was applied to statistically assess funnel plot asymmetry and the presence of small-study effects [27].

### 2.7. Ethical Considerations

Since this is secondary research, no ethical approval was required for this study, as it involved the analysis of previously published, de-identified data.

## 3. Results

### 3.1. Flow of Studies

**Figure 1:**
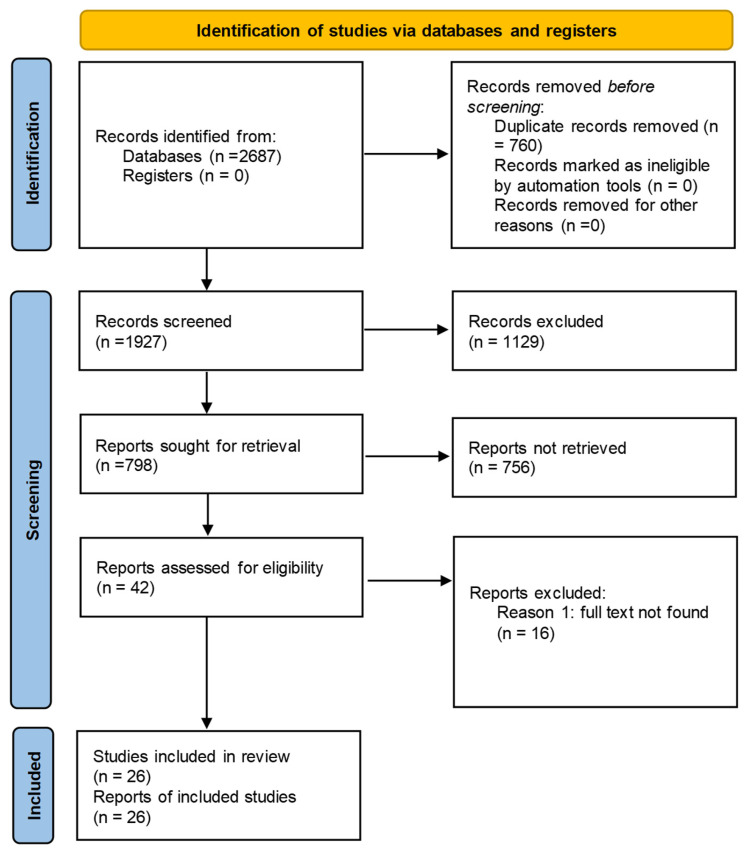
Flow of screening and identification of observational studies for review.

The study selection process for this meta-analysis is visually depicted in the flow diagram (Figure 1). The initial database search yielded 2687 records. After removing 760 duplicate entries, 1927 unique records remained. The subsequent screening of titles and abstracts resulted in the exclusion of 1129 records deemed irrelevant to the study, resulting in 798 potentially eligible records. Following this, 756 records were excluded for not meeting the eligibility criteria. Of the remaining 42 records, the full texts were reviewed, and 16 were excluded due to the full texts not being retrieved. A total of 26 studies met all the eligibility criteria and were included in the review and meta-analysis. The article selection adhered to the Preferred Reporting Items for Systematic Reviews and Meta-Analyses (PRISMA) criteria, (Checklists S1 and S2) [28]. 

### 3.2. Characteristics of Observational Studies Included in Meta-Analysis

Table 2 summarizes the characteristics and methodologies of the 26 included studies. The studies, published between 2001 and 2025, represent research conducted in various countries, including China (*n* = 12), Korea (*n* = 2), USA (*n* = 3), Turkey (*n* = 3), Italy (*n* = 3), Finland (*n* = 1), Singapore (*n* = 1), and Malaysia (*n* = 1). Sample sizes ranged widely, from a minimum of 43 participants in the study by Sonmez et al. [29] to a maximum of 8261 participants in the study by Lee et al. [30]. The minimum age of participants across studies was 18, while the maximum age reached 82, demonstrating a broad range of age groups included in the analysis. Regarding the specific sexual dysfunction outcomes reported, 6 studies (23.1%) focused solely on overall sexual dysfunction (SD), 10 studies (38.5%) examined only PE, and 24 studies (92.3%) investigated only ED. Furthermore, four studies (15.4%) each reported both SD and PE or ED and PE, and two studies (7.6%) included all three outcomes (SD, PE, and ED). Almost all studies used standard tools (e.g., NIH-CPSI, PEDT, IPSS, and IIEF-5) to assess SD, PE, or ED; however, only two studies assessed these outcomes using a self-reported questionnaire. This variation in assessed outcomes and the diversity of assessment tools used (with the NIH-CPSI being the most common) contribute to the heterogeneity observed in the meta-analysis.

### 3.3. CP/CPPS and Sexual Dysfunction

**Figure 2:**
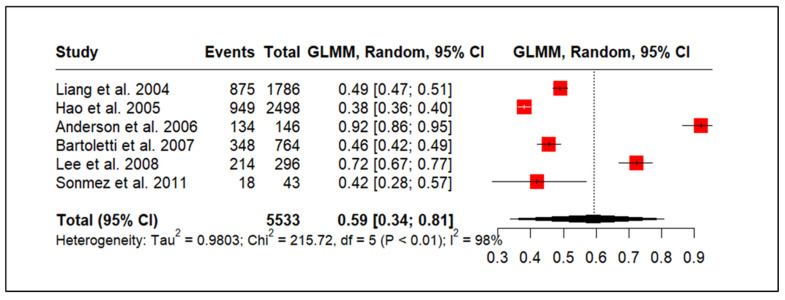
Overall sexual dysfunction prevalence in men with CP/CPPS [29,33,34,37,40,41].

Figure 2 presents a forest plot of the prevalence of the overall sexual dysfunction among men with CP/CPPS. Six studies, encompassing 5533 participants, assessed overall sexual dysfunction, including ejaculatory pain, decreased libido, erectile dysfunction, and ejaculatory dysfunction. The pooled proportion of men with CP/CPPS experiencing sexual dysfunction was estimated at 59%, with a wide 95% confidence interval (34% to 81%). This wide interval reflects the substantial heterogeneity observed among the included studies. The heterogeneity was statistically significant, as indicated by a tau-squared of 0.9803, a chi-squared statistic of 215.72 (5 degrees of freedom, *p* < 0.01), and an I-squared value of 98%. This high I-squared value suggests that the observed variability in sexual dysfunction prevalence across studies is primarily due to true differences between the study populations rather than chance.

### 3.4. CP/CPPS and Premature Ejaculation

**Figure 3:**
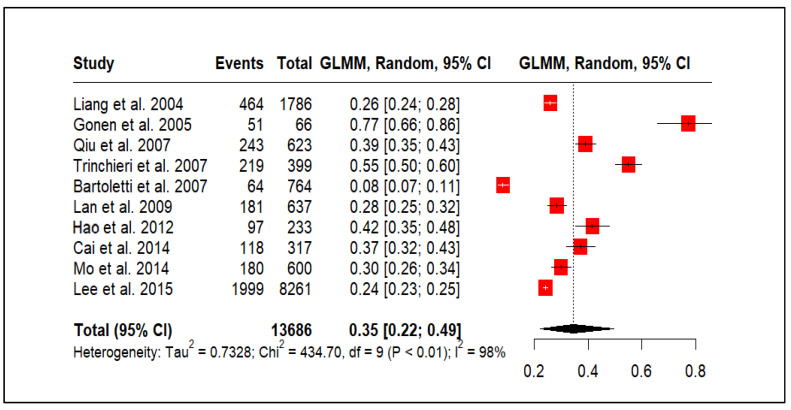
CP/CPPS and premature ejaculation prevalence [33,35,38,39,40,43,45,47,48,51].

Figure 3 shows the prevalence of premature ejaculation in men with CP/CPPS, based on 10 studies (*n* = 13,686). The pooled prevalence of PE was estimated to be 35%, with a wide 95% CI spanning from 22% to 49%. This broad confidence interval reflects the substantial heterogeneity observed across the included studies. Results indicated high heterogeneity (τ^2^ = 0.7328, χ^2^ = 434.70, df = 9, *p* < 0.01; I^2^ = 98%). This I-squared value indicates that the vast majority (98%) of the observed variation in premature ejaculation prevalence across the studies is likely attributable to real differences between the study populations rather than random chance.

### 3.5. CP/CPPS and Erectile Dysfunction

**Figure 4:**
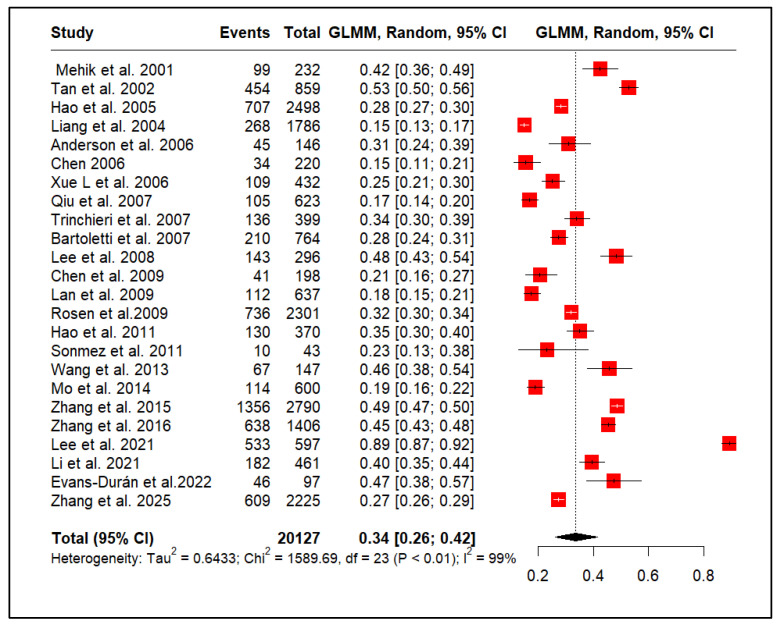
Erectile dysfunction prevalence in men with CP/CPPS [29,31,32,33,34,36,37,38,39,40,41,42,43,44,45,46,47,48,49,50,51,52,53,54].

Figure 4 presents a meta-analysis of 24 studies (20,127 participants) examining ED in men with CP/CPPS. The pooled prevalence was estimated at 34% (95% CI: 26–42%). However, considerable heterogeneity was observed across the studies, with a tau-squared of 0.6433, a highly significant chi-squared statistic of 1589.69 (df = 23, *p* < 0.01), and an extremely high I-squared value of 99%. This I-squared value indicates that an overwhelming majority (99%) of the observed variability in reported ED prevalence is likely due to true differences between the study populations rather than random chance.

### 3.6. Publication Bias

**Figure 5:**
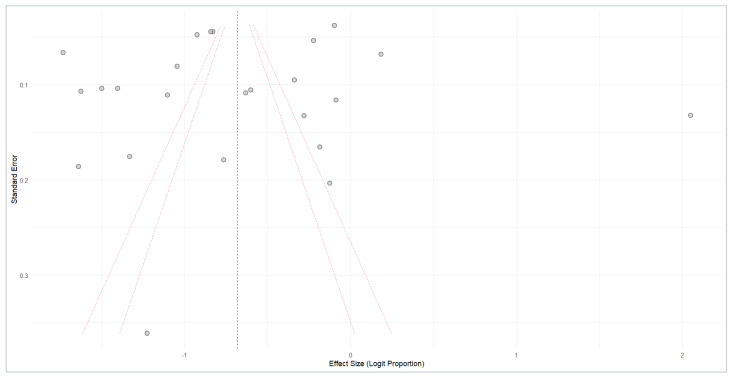
Funnel plot analysis of publication bias.

The publication bias in the sexual dysfunction meta-analysis is assessed in Figure 5 (funnel plot). The plot visualizes the relationship between the standard error and the logit-transformed proportion of sexual dysfunction for each study. While a symmetrical distribution is expected in the absence of bias, the observed plot exhibits some asymmetry, particularly with a scarcity of smaller studies in the bottom-left region, potentially indicating a lack of studies reporting lower prevalence. This visual suggestion of publication bias is further highlighted by the placement of several studies outside the inner (95% CI) and outer (99% CI) pseudo-confidence intervals (red dotted lines). Although the funnel plot visually suggested a potential publication bias, Egger’s regression test for funnel plot asymmetry did not yield a statistically significant result (t = −0.39, df = 22, *p*-value = 0.7034). This suggests that, statistically, there is no strong evidence of small-study effects or publication bias. Although the visual inspection hinted at a potential issue, the Egger’s test result tempers this concern, indicating that the apparent asymmetry might be due to chance or other factors unrelated to publication bias. Therefore, while the funnel plot warrants consideration, the lack of statistical support from Egger’s test suggests that publication bias is unlikely to be a substantial threat to the validity of the meta-analysis conclusions.

### 3.7. Study Quality and Risk of Bias

Supplemental Table S2 summarizes the quality assessment of the included studies, which were conducted using the STROBE checklist. This checklist contains 22 items related to the reporting quality of the observational studies, covering various sections, such as the title, abstract, introduction, methods, results, discussion, and other elements. Based on these 22 items of reporting criteria, the table shows that the quality of the included studies varies. A total quality score is provided for each study. Studies scoring between 14 and 19 are generally considered to be of moderate-to-good quality. In this review, 17 out of the 26 studies (approximately 65.4%) scored within this range, suggesting that a substantial proportion of the included studies had acceptable methodological rigor. However, the remaining nine studies (34.6%) scored below 14, indicating a higher risk of bias and potentially lower reliability of their findings.

### 3.8. Certainty of Evidence

As shown in Table 3, the certainty of evidence was rated as very low for sexual dysfunction due to a high risk of bias, very serious inconsistency, and serious imprecision arising from a wide confidence interval. For erectile dysfunction and premature ejaculation, the certainty of the evidence was rated as low. While these outcomes shared similar issues with risk of bias and high heterogeneity, their confidence intervals were narrower and based on larger sample sizes, so imprecision was not a major concern. No publication bias was detected for any outcome based on Egger’s test.

## 4. Discussion

This meta-analysis investigated the pooled prevalence of sexual dysfunction, including overall sexual dysfunction, ED, and PE, in men diagnosed with CP/CPPS. Our systematic search identified 26 observational studies encompassing over 20,000 participants, providing a comprehensive assessment of this prevalent comorbidity. The pooled prevalence of overall sexual dysfunction in men with CP/CPPS was 34%. When examining specific domains, we observed similar pooled prevalences for ED and PE, both at approximately 35% and 34%, respectively. These findings underscore the significant burden of sexual dysfunction among men diagnosed with CP/CPPS, highlighting the importance of recognizing and addressing these issues in clinical practice.

Our findings, consistent with earlier studies, indicate a high prevalence of sexual dysfunction within this population. A previous meta-analysis conducted by Chen et al. (2015) [15] examined the effect of CP/CPPS on erectile function and found a similar strong association. While they did not examine PE or overall sexual dysfunction in the same way we did, their conclusions about ED’s impact reinforce the notion that urological conditions like CP/CPPS are linked to sexual health issues. However, the author’s review was limited by the number and quality of studies, as the authors only included 10 studies, thereby influencing the power of the meta-analysis [15]. Another meta-analysis investigating the overall prevalence of sexual dysfunction in men suffering from chronic prostatitis/chronic pelvic pain syndrome (CP/CPPS) reported comparable findings. The study revealed that roughly one-third of men with CP/CPPS experience some type of sexual dysfunction. However, the authors did not consider including recently published studies published in the last decade [16]. Our study builds on these findings by providing a more granular analysis, specifically estimating the prevalence of ED and PE separately, which offers more clinically relevant information.

Similar prevalence estimates for overall sexual dysfunction, ED, and PE suggest that these domains are highly interconnected, and often, the mechanisms underlying this association are likely multifactorial, possibly involving shared pathophysiological pathways, psychological factors, and the impact of CP/CPPS symptoms on sexual function [55,56]. For instance, pelvic pain and urinary symptoms associated with CP/CPPS can directly interfere with sexual activity and contribute to psychological distress, both of which can negatively impact sexual function [57,58]. Furthermore, the chronic nature of CP/CPPS can lead to relationship strain and diminished quality of life, further exacerbating sexual difficulties [59].

Sexual dysfunction, particularly ED in men with CP/CPPS, is likely a result of a complex interplay of various factors rather than a singular cause [60,61,62]. While endocrine issues like hypogonadism do not appear to be directly linked to CP/CPPS, immune mediators and vascular factors may play a significant role [63,64]. Research indicates that men with CP/CPPS are more susceptible to peripheral arterial tone abnormalities, which may result from nitric oxide-mediated vascular endothelial dysfunction [64,65]. This can compromise the blood flow essential for achieving erections. Additionally, this reduced arterial inflow may be further worsened by extrinsic compression due to pelvic floor muscle spasms, a frequent occurrence in CP/CPPS patients [66]. Although true veno-occlusive disease is less probable in younger men with CP/CPPS, psychological factors like stress, anxiety, and catastrophizing are strongly implicated, which can significantly impact sexual function [67].

### Strengths and Limitations

This meta-analysis is strengthened by its comprehensive search strategy, the inclusion of a significant number of studies and participants, and the meticulous data extraction and quality assessment procedures employed. The random-effects model was utilized to account for the expected variability among studies. Nevertheless, several limitations must be recognized. The substantial heterogeneity observed across all analyses indicates differences in study populations, assessment tools, and definitions of sexual dysfunction. One key limitation of this meta-analysis is the variability in how ED and PE were defined and diagnosed across the included studies. Several studies relied on self-reported symptoms or lacked the use of standardized diagnostic criteria or validated tools as recommended by clinical guidelines. This methodological inconsistency may have contributed to an overestimation of prevalence rates and could partially explain the high heterogeneity observed in the pooled estimates. Further research is required to fully understand these variations and potential causes of heterogeneity. The possibility of publication bias, as suggested by the funnel plot and although statistically non-significant by Egger’s test, cannot be entirely dismissed. Additionally, the reliance on observational studies limits our ability to establish a causal relationship between CP/CPPS and the studied outcomes.

## 5. Conclusions

This meta-analysis confirms the high prevalence of sexual dysfunction in men with CP/CPPS. These findings underscore the importance of clinicians proactively assessing sexual function in this population and addressing these concerns as part of a comprehensive management approach. Further research is necessary to clarify the underlying mechanisms connecting CP/CPPS and sexual dysfunction. This will aid in the development of targeted interventions aimed at enhancing the sexual health and overall quality of life for individuals affected by this condition. Future studies should employ standardized assessment tools for sexual dysfunction and consider longitudinal designs to better understand the temporal relationship between CP/CPPS and sexual dysfunction. Moreover, there is a need for research to investigate the effectiveness of various treatment modalities for sexual dysfunction associated with CP/CPPS. This will help guide clinical practice and enhance patient outcomes.

### Implications for Future Research and Clinical Practice

This meta-analysis confirms the high prevalence of sexual dysfunction in men with CP/CPPS. These findings underscore the importance of clinicians proactively assessing sexual function in this population and addressing these concerns as part of a comprehensive management approach. Further research is necessary to clarify the underlying mechanisms linking CP/CPPS and sexual dysfunction, which will facilitate the development of targeted interventions to enhance the sexual health and overall quality of life in affected individuals.

Future studies should focus on several key areas. First, the use of standardized and validated assessment tools for evaluating sexual dysfunction is essential to ensure comparability across studies. Second, longitudinal research designs are needed to elucidate the temporal relationship and potential causality between CP/CPPS and the onset or progression of sexual dysfunction. Third, there is a critical need to evaluate the effectiveness of various treatment modalities—including pharmacological, behavioral, and multimodal interventions—for managing sexual dysfunction in the context of CP/CPPS. In addition, future research should explore patient-centered outcomes, including satisfaction with care and quality of life, to better inform clinical decision-making. Finally, subgroup analyses by age, symptom duration, or severity may help identify individuals at greater risk and inform personalized treatment strategies.

## Figures and Tables

**Table 1:** Example of search strategy using the PICO framework.

PICO Element	Search Terms/Keywords	Variations/Combinations	Example (PubMed)
P (Population/Participants)	Men	Male, Males	PubMed: “male” [MeSH Terms] OR “men” [Title/Abstract]
	Chronic Prostatitis	Chronic Bacterial Prostatitis, Chronic Pelvic Pain Syndrome, CP/CPPS, Prostatitis-like syndrome	PubMed: “prostatitis” [MeSH Terms] OR “chronic prostatitis” [Title/Abstract] OR “CPPS” [Title/Abstract]
I (Intervention/Exposure)	(Not directly applicable for prevalence studies. Focus is on the condition/problem.)	N/A	N/A
C (Comparison/Control)	(Often not applicable for prevalence studies. Could be men without prostatitis if comparing prevalence rates, but our focus is on men with prostatitis.)	Men without CP/CPPS (if a comparative prevalence is sought)	PubMed: “healthy men” [Title/Abstract] (Use with Population terms if comparing)
O (Outcome)	Sexual Dysfunction	Erectile Dysfunction, Premature Ejaculation, Ejaculatory Dysfunction, Decreased Libido, Painful Ejaculation, Sexual Problems, Sexual Health, Sexual Function	PubMed: “sexual dysfunction” [MeSH Terms] OR “erectile dysfunction” [MeSH Terms] OR “premature ejaculation” [MeSH Terms] OR “sexual health” [MeSH Terms]
Scope	Prevalence	Frequency, Occurrence, Epidemiology, Survey, Questionnaire, Cross-sectional	PubMed: “prevalence” [MeSH Terms] OR “epidemiology” [MeSH Terms] OR “survey” [Title/Abstract]

**Table 2:** Characteristics of observational studies included in the meta-analysis.

Author	Year	Country	*n*	Age	Tools	Outcomes	SD	ED	PE
Mehik et al., 2001 [31]	2001	Finland	232	20–59	Self-reported	ED	NR	42.5%	NR
Tan et al., 2002 [32]	2002	Singapore	859	43.14	NIH-CPSI and IIEF-5	ED	NR	52.9%	NR
Liang et al., 2004 [33]	2004	China	1786	20–59	NIH-CPSI and IIEF-5	SD and ED	49.0%	15.0%	26.0%
Hao et al., 2005 [34]	2005	China	2498	20–59	NIH-CPSI and IIEF-5	SD and ED	38.0%	28.3%	NR
Gonen et al., 2005 [35]	2005	Turkey	66	21–55	NIH-CPSI	PE	NR	NR	77.3%
Xue L et al., 2006 [36]	2006	China	432	22–45	NIH-CPSI and IIEF-5	ED	NR	25.2%	NR
Anderson et al., 2006 [37]	2006	USA	146	18–77	NIH-CPSI and PPSS	SD and ED	92.0%	31%	NR
Qiu et al., 2007 [38]	2007	China	623	18–57	CISFPE and IIEF-5	ED and PE	NR	16.9%	39.0%
Trinchieri et al., 2007 [39]	2007	Italy	399	<50	NIH-CPSI	ED and PE	NR	34%	55.0%
Bartoletti et al., 2007 [40]	2007	Italy	764	25–50	NIH-CPSI and IIEF-5	SD, ED, and PE	45.5%	27.5%	8.4%
Lee et al., 2008 [41]	2008	Malaysia	296	20–69	NIH-CPSI and IIEF-5	SD and ED	72.3%	48.3%	NR
Chen et al., 2009 [42]	2009	China	198	20–59	NIH-CPSI and IIEF-5	ED	NR	20.7%	NR
Lan et al., 2009 [43]	2009	China	637	25–61	CISFPE and IIEF-5	ED and PE	NR	17.6%	28.4%
Rosen et al., 2009 [44]	2009	USA	2301	30–79	NIH-CPSI, IPSS, and IIEF-5	ED	NR	32.0%	NR
Hao et al., 2011 [45]	2011	China	370	15–60	NIH-CPSI and IIEF-5	ED	NR	35.1%	NR
Sonmez et al., 2011 [29]	2011	Turkey	43	22–48	NIH-CPSI and IIEF	SD and ED	41.9%	23.3%	NR
Wang et al., 2013 [46]	2013	China	147	18–64	NIH-CPSI and IIEF-5	ED	NR	45.8%	NR
Cai et al., 2014 [47]	2014	Italy	317	33.8 ± 5.1	NIH-CPSI and PEDT	PE	NR	NR	37.2%
Mo et al., 2014 [48]	2014	China	600	28.95 ± 4.98	NIH-CPSI and IIEF-5	ED and PE	NR	19.0%	30.0%
Lee et al., 2015 [30]	2015	Korea	8261	50.4 ± 5.5	NIH-CPSI and IIEF-5	PE	NR	NR	24.2%
Zhang et al., 2015 [49]	2015	China	2790	40.10 ± 0.58	NIH-CPSI and IIEF-5	ED	NR	48.6%	NR
Zhang et al., 2016 [50]	2016	China	1406	32.18 (18–60)	NIH-CPSI and IIEF-5	ED	NR	45.4%	NR
Lee et al., 2021 [51]	2021	Republic of Korea	597	50.9 ± 5.6	NIH-CPSI, PEDT, and IIEF-5	ED	NR	89.3%	NR
Li et al., 2021 [52]	2021	China	461	33.86 ± 8.36	NIH-CPSI, PEDT, and IIEF-6	ED	NR	39.5%	NR
Evans-Durán et al., 2022 [53]	2022	Multi-country	97	44.22 ±11.25	NIH-CPSI and IIEF-5	ED	NR	47.4%	NR
Zhang et al., 2025 [54]	2025	USA	2225	40–80	Self-reported	ED	NR	27.5%	NR

SD: Sexual dysfunction. ED: Erectile dysfunction. PE: Premature ejaculation. NR: Not reported. USA: United States of America; NIH-CPSI—National Institutes of Health Chronic Prostatitis Symptom Index; IIEF-5—International Index of Erectile Function—5-item version; PPSS—Pain, Urgency, and Frequency Prostatitis Symptom Score; IPSS—International Prostate Symptom Score.

**Table 3:** GRADE summary of certainty for outcomes.

Outcome	No. of Studies	Participants	Risk of Bias	Inconsistency (I^2^)	Indirectness	Imprecision	Publication Bias	Certainty of Evidence
Sexual Dysfunction	6	5533	Serious	Very serious (98%)	Not serious	Serious	Not detected	Very Low
Erectile Dysfunction	10	13,686	Serious	Very serious (98%)	Not serious	Not serious	Not detected	Low
Premature Ejaculation	24	20,127	Serious	Very serious (99%)	Not serious	Not serious	Not detected	Low

Risk of Bias (serious): Many included studies were observational and assessed as having either a high risk of bias or some concerns. Inconsistency (very serious): Extremely high heterogeneity across studies (I^2^ > 98%) was observed for all three outcomes, indicating a substantial variation in study results. Indirectness (not serious): All studies directly addressed the population (men with CP/CPPS) and outcomes of interest (types of sexual dysfunction), with no major concerns regarding applicability. Imprecision (serious): For sexual dysfunction, the 95% confidence interval ranged widely from 34% to 81%, suggesting uncertainty in the exact prevalence and crossing thresholds that could impact clinical decision-making. Imprecision (not serious): For erectile dysfunction and premature ejaculation, the confidence intervals were relatively narrow and based on large sample sizes (>10,000 participants), making the prevalence estimates more stable and reliable. Publication Bias (not detected): Egger’s test did not suggest the presence of publication bias for any of the outcomes evaluated.

## Data Availability

No new data were generated for this study. All data used in this study were already published and freely available online.

## Supplementary Materials

The following supporting information can be downloaded at: https://www.mdpi.com/article/10.3390/medicina61061110/s1, Table S1: Final Search Strategy for Each Database; Table S2. Finding related to Risk bias of observational studies included in meta-analysis (*n* = 26). Checklist S1: PRISMA 2020 abstract checklist; Checklist S2: PRISMA 2020 checklist.

## Abbreviations

The following abbreviations are used in this manuscript:

CP	Chronic prostatitis
CPPS	Chronic Pelvic Pain Syndrome
EAU	European Association of Urology
PE	Premature Ejaculation
ED	Erectile Dysfunction
MOOSE	Meta-analysis of Observational Studies in Epidemiology
PICO	Population, Intervention, Comparison, and Outcome
