# Supplementary material for: Prevalence of Sexual Dysfunction with Chronic Prostatitis/Chronic Pelvic Pain Syndrome (CP/CPPS): An Updated Systematic Review and Meta-Analysis
Source: Medicina (Kaunas). 2025 Jun 19;61(6):1110. doi: 10.3390/medicina61061110 (PMC12195409; doi:10.3390/medicina61061110)
Supplement: Supplementary file 1 [file medicina-61-01110-s001.zip › medicina-3629361-supplementary.pdf]

Section and Topic	Item #	Checklist item	Reported (Yes/No)
**TITLE**			
Title	1	Identify the report as a systematic review.	Line #2-3, page 1
**BACKGROUND**			
Objectives	2	Provide an explicit statement of the main objective(s) or question(s) the review addresses.	Line #16-18, page 1
**METHODS**			
Eligibility criteria	3	Specify the inclusion and exclusion criteria for the review.	Line #21-22, page 1
Information sources	4	Specify the information sources (e.g., databases and registers) used to identify studies and the date when each was last searched.	Line #19-20, page 1
Risk of bias	5	Specify the methods used to assess the risk of bias in the included studies.	Line #25, page 1
Synthesis of results	6	Specify the methods used to present and synthesize results.	Line #22-25, page 1
**RESULTS**			
Included studies	7	Give the total number of included studies and participants and summarize the relevant characteristics of the studies.	Line #26-27, page 1
Synthesis of results	8	Present the results for the main outcomes, preferably indicating the number of included studies and participants for each. If a meta-analysis was performed, report the summary estimate and confidence/credible interval. If comparing groups, indicate the direction of the effect (i.e., which group is favored).	Line #27-33, page 1
**DISCUSSION**			
Limitations of evidence	9	Provide a brief summary of the limitations of the evidence included in the review (e.g., study risk of bias, inconsistency, and imprecision).	Line #35-36, page 1
Interpretation	10	Provide a general interpretation of the results and important implications.	Line #36-37, page 1
**OTHER**			
Funding	11	Specify the primary source of funding for the review.	

Section and Topic	Item #	Checklist item	Reported (Yes/No)
Registration	12	Provide the register name and registration number.	N/A

From: Page MJ, McKenzie JE, Bossuyt PM, Boutron I, Hoffmann TC, Mulrow CD et al. The PRISMA 2020 statement: an updated guideline for reporting systematic reviews. BMJ 2021;372:n71. doi: 10.1136/bmj.n71. This work is licensed under CC BY 4.0. To view a copy of this license, visit https://creativecommons.org/licenses/by/4.0/

Section and Topic	Item #	Checklist item	Location where item is reported
**TITLE**			
Title	1	Identify the report as a systematic review.	Line #2-3, page 1
**ABSTRACT**			
Abstract	2	See the PRISMA 2020 for Abstracts checklist.	Supplementary material S1
**INTRODUCTION**			
Rationale	3	Describe the rationale for the review in the context of existing knowledge.	Line #71-79, page 2
Objectives	4	Provide an explicit statement of the objective(s) or question(s) the review addresses.	Line #82-83, page 1
**METHODS**			
Eligibility criteria	5	Specify the inclusion and exclusion criteria for the review and how studies were grouped for the syntheses.	Line #113-131, page 4
Information sources	6	Specify all databases, registers, websites, organizations, reference lists, and other sources searched or consulted to identify studies. Specify the date when each source was last searched or consulted.	Line #92-93, page 3
Search strategy	7	Present the full search strategies for all databases, registers, and websites, including any filters and limits used.	Line #93-110, page 2-4
Selection process	8	Specify the methods used to decide whether a study met the inclusion criteria of the review, including how many reviewers screened each record and each report retrieved, whether they worked independently, and, if applicable, details of the automation tools used in the process.	Line #112-134, page 4
Data collection process	9	Specify the methods used to collect data from reports, including how many reviewers collected data from each report, whether they worked independently, any processes for obtaining or confirming data from study investigators, and, if applicable, details of the automation tools used in the process.	Line #136-149, page 4
Data items	10a	List and define all outcomes for which data were sought. Specify whether all results that were compatible with each outcome domain in each study were sought (e.g., for all measures, time points, analyses), and if not, the methods used to decide which results to collect.	Line #92-110, page 3
	10b	List and define all other variables for which data were sought (e.g., participant and intervention characteristics and funding sources). Describe any assumptions made about any missing or unclear information.	Line #92-110, page 3
Study risk of bias assessment	11	Specify the methods used to assess risk of bias in the included studies, including details of the tool(s) used, how many reviewers assessed each study, whether they worked independently, and, if applicable, details of the automation tools used in the process.	Line #172-197, page 5
Effect measures	12	For each outcome, specify the effect measure(s) (e.g., risk ratio and mean difference) used in the synthesis or presentation of the results.	Line #172-197, page 5
Synthesis methods	13a	Describe the processes used to decide which studies were eligible for each synthesis (e.g., tabulating the study intervention characteristics and comparing against the planned groups for each synthesis (item #5)).	Line #172-197, page 5

Section and Topic	Item #	Checklist item	Location where item is reported
	13b	Describe any methods required to prepare the data for presentation or synthesis, such as the handling of missing summary statistics or data conversions.	Line #172-197, page 5
	13c	Describe any methods used to tabulate or visually display the results of individual studies and syntheses.	Line #172-197, page 5
	13d	Describe any methods used to synthesize results and provide a rationale for the choice(s). If meta-analysis was performed, describe the model(s), method(s) to identify the presence and extent of statistical heterogeneity, and software package(s) used.	Line #172-197, page 5
	13e	Describe any methods used to explore possible causes of heterogeneity among study results (e.g., subgroup analysis and meta-regression).	Line #173-176, page 5
	13f	Describe any sensitivity analyses conducted to assess the robustness of the synthesized results.	Line #172-197, page 5
Reporting bias assessment	14	Describe any methods used to assess the risk of bias due to missing results in a synthesis (arising from reporting biases).	Line #178-187, page 5
Certainty assessment	15	Describe any methods used to assess certainty (or confidence) in the body of evidence for an outcome.	Line #185-197, page 5
**RESULTS**			
Study selection	16a	Describe the results of the search and selection process, from the number of records identified in the search to the number of studies included in the review, ideally using a flow diagram.	Line #200-211, page 6
	16b	Cite studies that might appear to meet the inclusion criteria, but which were excluded, and explain why they were excluded.	Line #202-207, page 5
Study characteristics	17	Cite each included study and present its characteristics.	Table 2, page 7
Risk of bias in studies	18	Present assessments of risk of bias for each included study.	Line # 286-321, page 12-13
Results of individual studies	19	For all outcomes, present, for each study, the following: (a) summary statistics for each group (where appropriate) and (b) an effect estimate and its precision (e.g., confidence/credible interval), ideally using structured tables or plots.	Line # 200-285, page 5-12
Results of syntheses	20a	For each synthesis, briefly summarize the characteristics and risk of bias among contributing studies.	Line # 286-321, page 12-13
	20b	Present the results of all statistical syntheses conducted. If meta-analysis was performed, present for each the summary estimate and its precision (e.g., confidence/credible interval) and measures of statistical heterogeneity. If comparing groups, describe the direction of the effect.	Line # 200-285, page 5-12

Section and Topic	Item #	Checklist item	Location where item is reported
	20c	Present the results of all the investigations of possible causes of heterogeneity among study results.	Line # 232-267, page 5-12
	20d	Present the results of all sensitivity analyses conducted to assess the robustness of the synthesized results.	Line # 232-267, page 5-12
Reporting biases	21	Present assessments of risk of bias due to missing results (arising from reporting biases) for each synthesis assessed.	Line # 268-285, page 12-13
Certainty of evidence	22	Present assessments of certainty (or confidence) in the body of evidence for each outcome assessed.	-
**DISCUSSION**			
Discussion	23a	Provide a general interpretation of the results in the context of other evidence.	Line # 374-381, page 14-15
	23b	Discuss any limitations of the evidence included in the review.	Line # 323-381, page 15
	23c	Discuss any limitations of the review processes used.	Line # 323-381, page 15
	23d	Discuss implications of the results for practice, policy, and future research.	Line # 389-394, page 15-16
**OTHER INFORMATION**			
Registration and protocol	24a	Provide registration information for the review, including the register name and registration number, or state that the review was not registered.	N/A
	24b	Indicate where the review protocol can be accessed, or state that a protocol was not prepared.	N/A
	24c	Describe and explain any amendments to the information provided at registration or in the protocol.	N/A
Support	25	Describe the sources of financial or non-financial support for the review, and the role of the funders or sponsors in the review.	Line # 401, page 16
Competing interests	26	Declare any competing interests of review authors.	Line # 408, page 16
Availability of data, code, and	27	Report which of the following are publicly available and where they can be found: template data collection forms; data extracted from included studies; data used for all analyses; analytic code; any other materials used in the review.	Line # 404-405, page 16

Section and Topic	Item #	Checklist item	Location where item is reported
other materials			

From: Page MJ, McKenzie JE, Bossuyt PM, Boutron I, Hoffmann TC, Mulrow CD et al. The PRISMA 2020 statement: an updated guideline for reporting systematic reviews. BMJ 2021;372:n71. doi: 10.1136/bmj.n71. This work is licensed under CC BY 4.0. To view a copy of this license, visit https://creativecommons.org/licenses/by/4.0/

Supplemental Table S1: Final search strategy for each database.

**Database**	**Search Strategy**
**PubMed**	("male"[MeSH Terms] OR "men"[Title/Abstract] OR "males"[Title/Abstract]) AND ("prostatitis"[MeSH Terms] OR "chronic prostatitis"[Title/Abstract] OR "chronic pelvic pain syndrome"[Title/Abstract] OR "CPPS"[Title/Abstract]) AND ("sexual dysfunction"[MeSH Terms] OR "erectile dysfunction"[MeSH Terms] OR "premature ejaculation"[MeSH Terms] OR "sexual health"[MeSH Terms] OR "ejaculatory dysfunction"[Title/Abstract] OR "decreased libido"[Title/Abstract] OR "painful ejaculation"[Title/Abstract] OR "sexual problems"[Title/Abstract] OR "sexual function"[Title/Abstract]) AND ("prevalence"[MeSH Terms] OR "epidemiology"[MeSH Terms] OR "survey"[Title/Abstract] OR "frequency"[Title/Abstract] OR "cross-sectional"[Title/Abstract])
**Embase**	('male'/exp OR men:ti,ab OR males:ti,ab) AND ('prostatitis'/exp OR 'chronic prostatitis':ti,ab OR 'chronic pelvic pain syndrome':ti,ab OR CPPS:ti,ab) AND ('sexual dysfunction'/exp OR 'erectile dysfunction'/exp OR 'premature ejaculation'/exp OR 'sexual health'/exp OR 'ejaculatory dysfunction':ti,ab OR 'decreased libido':ti,ab OR 'painful ejaculation':ti,ab OR 'sexual problems':ti,ab OR 'sexual function':ti,ab) AND ('prevalence'/exp OR 'epidemiology'/exp OR survey:ti,ab OR frequency:ti,ab OR 'cross-sectional':ti,ab)
**Web of Science**	(TS=(male OR men OR males)) AND (TS=("chronic prostatitis" OR "chronic pelvic pain syndrome" OR CPPS OR prostatitis)) AND (TS=("sexual dysfunction" OR "erectile dysfunction" OR "premature ejaculation" OR "sexual health" OR "ejaculatory dysfunction" OR "decreased libido" OR "painful ejaculation" OR "sexual problems" OR "sexual function")) AND (TS=(prevalence OR epidemiology OR survey OR frequency OR "cross-sectional"))
**Google Scholar**	allintitle: ("chronic prostatitis" OR "chronic pelvic pain syndrome" OR CPPS) AND ("sexual dysfunction" OR "erectile dysfunction" OR "premature ejaculation" OR "sexual health" OR "ejaculatory dysfunction" OR "decreased libido" OR "painful ejaculation" OR "sexual problems" OR "sexual function") AND (prevalence OR epidemiology OR survey OR frequency OR "cross-sectional")

Supplemental Table S2. Findings related to the risk of bias of observational studies included in meta-analysis (n=26).

**Authors**	**STROBE checklist Items**																						**Total score**
	**1**	**2**	**3**	**4**	**5**	**6**	**7**	**8**	**9**	**10**	**11**	**12**	**13**	**14**	**15**	**16**	**17**	**18**	**19**	**20**	**21**	**22**	
Mehik et al. 2001	1	1	1	1	1	1	1	1	0	0	1	0	1	0	1	1	0	1	0	0	0	1	14
Tan et al. 2002	1	1	1	1	1	1	1	1	0	0	1	0	1	1	1	0	0	1	0	0	0	0	13
Liang et al. 2004	1	1	1	1	1	1	1	1	0	0	0	0	1	1	1	1	1	0	0	0	0	0	13
Hao et al. 2005	0	1	1	0	1	1	1	1	1	1	0	0	1	1	1	0	0	1	1	1	0	0	14
Gonen et al. 2005	1	1	1	0	1	1	1	1	1	1	1	0	1	1	1	1	0	1	0	1	0	0	16
Xue L et al. 2006	1	1	1	1	1	1	1	1	1	1	1	0	1	1	1	0	0	1	0	1	0	1	17
Anderson et al. 2006	1	1	1	1	1	1	1	1	1	1	0	0	1	1	1	0	1	1	1	1	0	0	17
Qiu et al. 2007	0	1	1	0	1	1	1	1	1	1	0	0	1	1	1	0	0	1	1	1	0	0	14
Trinchieri et al. 2007	1	1	1	1	1	1	1	1	0	0	1	0	1	1	1	0	1	1	0	1	0	1	16
Bartoletti et al. 2007	1	1	1	0	1	1	1	1	1	1	0	0	1	1	1	0	1	1	1	1	0	0	16
Lee et al. 2008	1	1	1	0	1	1	1	1	1	1	1	1	1	1	1	0	0	1	1	1	1	0	18
Chen et al. 2009	1	1	1	1	1	1	1	1	1	1	1	0	1	1	1	0	1	1	1	1	0	1	19
Lan et al. 2009	1	1	0	0	1	1	1	1	1	1	0	0	1	1	1	0	1	1	1	1	1	0	16
Rosen et al. 2009	1	1	1	1	1	0	1	1	0	0	1	1	1	1	1	1	1	1	1	0	0	0	16
Hao et al. 2011	1	1	1	0	1	1	1	1	0	1	1	0	1	1	1	0	1	1	0	1	0	1	16
Sonmez et al. 2011	1	1	1	1	1	1	1	1	0	1	1	0	1	1	1	0	0	1	0	1	0	1	16
Wang et al. 2013	1	1	1	1	1	1	1	1	1	1	1	0	1	1	1	0	1	1	0	1	0	0	16
Cai et al. 2014	1	1	1	1	1	1	1	1	0	1	1	0	1	1	1	0	1	1	1	1	1	0	18

Mo et al. 2014	1	1	1	1	1	1	1	1	0	0	1	0	1	1	1	1	0	1	1	0	1	0	16
Lee et al. 2015	1	1	1	0	1	1	1	1	0	0	1	0	1	1	1	1	0	1	1	0	0	1	15
Zhang et al. 2015	1	1	1	1	1	1	1	1	0	0	1	0	1	1	1	1	0	1	1	1	0	1	17
Zhang et al. 2016	1	1	1	1	1	1	1	1	0	0	1	0	1	1	1	1	1	1	1	0	1	1	18
Lee et al. 2021	1	1	1	1	1	1	1	1	0	0	1	0	1	1	1	1	0	1	1	0	0	0	15
Li et al. 2021	1	1	1	1	1	1	1	1	0	0	1	0	1	1	1	0	0	1	0	0	0	1	14
Evans-Durán et al. 2022	1	1	1	0	1	1	1	1	0	0	1	0	1	1	1	1	1	1	1	1	1	0	17
Zhang et al. 2025	1	1	1	1	1	1	1	1	0	0	1	1	1	1	1	1	1	1	1	0	1	1	19

1)Title and Abstract: Clear, informative, and balanced summary of the study.

2) Background/Rationale: Context and rationale for the study clearly presented in the introduction.

3) Objectives: Specific study objectives clearly stated in the introduction.

4) Study Design: Design of the study accurately described early in the paper.

5) Setting: Description of the study setting, locations, and relevant dates.

6) Participants: Clear description of eligibility criteria, participant selection sources, and methods.

7) Definitions: Clear definitions of all variables: diagnostic criteria, outcomes, exposures, predictors, potential confounders, and effect modifiers.

8) Data Sources/Measurement: Description of data sources and measurement methods for each variable.

9) Bias: Description of any efforts to address potential sources of bias.

10) Study Size: Clear explanation of how the study sample size was determined.

11) Statistical Methods: Description of all statistical methods, including those for controlling confounding.

12) Sampling Strategy: Description of analytical methods that account for the sampling strategy.

13) Study Population: Reporting of the number of individuals in the study.

14) Participant Characteristics: Clear description of study characteristics and number of participants.

15) Outcome Data: Reporting of the number of outcome events or summary measures.

16) Adjusted Estimates: Reporting of confounder-adjusted risk estimates and their 95% confidence intervals.

17) Subgroup/Interaction Analyses: Reporting of subgroup and interaction analyses.

18) Key Results: Summary of key results in relation to the study objectives.

19) Limitations: Discussion of the study's limitations.

20) Interpretation: Cautious overall interpretation of the study results.

21) Generalizability: Discussion of the study's generalizability (external validity).

22) Funding: Description of funding sources and the role of funders.
